# First-principles structural, elastic and optoelectronics study of sodium niobate and tantalate perovskites

**DOI:** 10.1038/s41598-022-26250-7

**Published:** 2022-12-15

**Authors:** Shaukat Ali Khattak, Saikh Mohammad Wabaidur, Md Ataul Islam, Mudasser Husain, Irfan Ullah, Syed Zulfiqar, Gul Rooh, Nasir Rahman, Muhammad Salman Khan, Gulzar Khan, Tahirzeb Khan, Benabdellah Ghlamallah

**Affiliations:** 1 Department of Physics, Abdul Wali Khan University Mardan, Mardan, 23200 Pakistan; 2 Chemistry Department, College of Science, King Saud University, Riyadh, 11451 Saudi Arabia; 3 Division of Pharmacy and Optometry, School of Health Sciences, Faculty of Biology, Medicine and Health, University of Manchester, Manchester, UK; 4 Department of Physics, University of Lakki Marwat, Lakki Marwat, 28420 Pakistan; 5 Department of Physics, Faculty of Matter Sciences, University of Tiaret, Laboratory of Physical Engineering, Tiaret, Algeria

**Keywords:** Materials science, Physics

## Abstract

The intensified quest for efficient materials drives us to study the alkali (Na)-based niobate (NaNbO_3_) and tantalate (NaTaO_3_) perovskites while exploiting the first-principles approach based on density functional theory, coded within WIEN2K. While using the Birch Murnaghan fit, we find these materials to be stable structurally. Similarly, the ab-initio molecular dynamics simulations (AIMD) at room temperature reveals that the compounds exhibit no structural distortion and are stable at room temperature. By using the recommended modified Becke–Johnson potential, we determine the electronic characteristics of the present materials providing insight into their nature: they are revealed to be indirect semiconductors with the calculated bandgaps of 2.5 and 3.8 eV for NaNbO_3_ and NaTaO_3_, respectively. We also determine the total and partial density of states for both materials and the results obtained for the bandgap energies of these materials are consistent with those determined by the band structure. We find that both compounds exhibit transparency to the striking photon at low energy and demonstrate absorption and optical conduction in the UV region. The elastic study shows that these compounds are mechanically stable, whereas NaNbO_3_ exhibits stronger ability to withstand compressive as well as shear stresses and resists change in shape while NaTaO_3_ demonstrates weaker ability to resist change in volume. We also find that none of the compound is perfectly isotropic and NaNbO_3_ and NaTaO_3_ are ductile and brittle in nature, respectively. By studying the optical properties of these materials, we infer that they are promising candidates for applications in optoelectronic devices. We believe that this report will invoke the experimental studies for further investigation.

## Introduction

Due to potential applications in photovoltaic cells^1^, lasers^2^, X-ray detectors^3^, light-emitting nanoantenna^4^, tunable and efficient light-emitting diodes^5^, 3-D nano-printing^6^ and ultra-high resolution color displays and multilevel anticounterfeiting^7^, superconductors, multiferroics, batteries, fuel cells, photovoltaic electrodes, catalysts, resistive switches, and sensing materials^8–10^, perovskites materials, have garnered considerable attention. They have the structure of CaTiO_3_, which was reported by Dick Megaw with X-ray diffractions (XRD)^11^.

A formula of ABX_3_ represents the perovskite where A and B are cations with A typically of a larger radius than the B and X denoting an anion. Exhibiting wonderful optoelectronic characteristics, the halide perovskites, an important section of the perovskites, have been under investigation for thermoelectric, memory, and artificial synapse applications^12^. They have shown large absorption coefficients, charge carriers with high mobility^13^, and carrier diffusing relatively more^14^. While demonstrating these properties and given the fact that they possess more-than-25-% power conversion efficiency^15^, they are potential photovoltaic candidates^1,16,17^. Since halide perovskites are highly versatile, their bandgaps are engineerable while altering the composition of the inorganic framework, by the selectivity of cation (both organic and inorganic), stoichiometric changes, structure with different layers^18–21^, and nanoparticles^22^. Since halide perovskites are interesting optically, electrically, and magnetically, they are good candidates for optoelectronic applications. Similarly, the oxide perovskites have drawn great attention thanks to their seamless availability and distinctive properties which are considerably tailorable, thus making them suitable for numerous applications^23^. They are represented by ABO_3_, A and B indicate cations. Generally, the radius of the cation residing on site A is larger than that of the cation located on site B. These cations correspond to a divalent (or monovalent) metal bonding with 12 O anions and a tetra (or pentavalent) atom, making bonds with 6 O anions. Their properties have widely been investigated. Muhammad Saeed et al. studied the alkali niobates, i.e., ANbO_3_, structurally and opto-electronically, and found that these niobates are semiconductors exhibiting indirect bandgaps^24^. SrTMO_3_ (TM = Mn, Fe, Co, Tc, Ru, Rh, Re, Os, Ir) were studied electronically, elastically and magnetically by Uzma Qazi et al. where they were revealed to be anisotropic, ductile, and mechanically stable^25^. Computationally investigated by Somia et al., AMoO_3_ (A = Ca, Sr, and Ba) oxide perovskites have been found to exhibit metallic nature, para-magnetism, mechanical stability, ductility, and anisotropy^26^. Shahid Mehmood et al. studied the SrFeO_3_ perovskites oxides electronically and magnetically where they (SrFeO_3_) demonstrated ferromagnetism and metallic nature^27^, although their previous investigation has revealed them to be helicoidal anti-ferromagnet^28^. Akbar Ali et al. investigated the BiFeO_3_ and BaTiO_3_ perovskites oxides^42^. L.G. Tejuca and his coworker investigated optical, electric, and magnetic characteristics of perovskite-type oxides^29,30^. Smyth et al. have thoroughly studied the optoelectronic characteristics of the Ca_2_Fe_2_O_5_ perovskites where they inferred that the studied perovskites can be efficient for photocatalysis and photosynthesis^29^. Nancy et al. have studied the brownmillerite perovskites where they investigated the influence of vacancies on the mechanical properties of these perovskites^43^. While studying the La_2_Ni_2_O_5_ metallic perovskites, Synagues et al. found that these perovskites are metallic and stable which is predominantly due to the substitution of cations on cites A and B partially, leading to the remarkable optical characteristics^31^. J. Sfeir et al. studied the CaCr_2_O_4_ and CaZrO_3_ perovskites where they looked for fuel oxidation by the anode contribution^31^. Pen˜a and Fierro et al. investigated the utility of oxide perovskites for applications in surface chemistry and heterogenous catalysis by determining the physical and optoelectronic characteristics for potential optoelectronic applications^32^. Since the experimental investigations require resources (materials and experimental setups) as well as are time consuming, computational study of the materials is a promising approach to explore the new materials for wide-ranging applications. This can not only validate the already-carried-out experimental studies, but also persuade the experimentalists to investigate the simulated new materials.

In this work, we study alkali (Na)-based niobate (NaNbO_3_) and tantalate (NaTaO_3_) perovskites while exploiting the density functional theory (DFT) and the full potential linearized augmented plane wave (FP-LAPW), coded in WIEN2K. We believe the optical and mechanical properties of these materials haven’t been studied theoretically. We determine their structural, optoelectronic, and elastic properties. The structural study suggests that these compounds are stable. While using the mBJ method, the present perovskites exhibit an indirect bandgap semiconducting nature where NaNbO_3_ and tantalate NaTaO_3_ have 2.5 eV and 3.8 eV bandgap energy. Both compounds are mechanically stable with NaNbO_3_ exhibiting stronger ability of withstanding compressive and shear stresses and opposing change in shape and NaTaO_3_ demonstrating weaker ability of resisting change in volume. While none of the compound is perfectly isotropic, NaNbO_3_ and NaTaO_3_ exhibit ductility and brittleness, respectively. The computed optical properties demonstrate that with distinctive properties they are potentially useful for UV-devices applications.

## Computational details

Using the full-potential linearized augmented-plane wave (FP-LAPW) method^33,34^, coded in the WIEN2K^35^, was exploited for the solution of Kohn Sham equations. This is a well-considered method, developed while using the DFT, for the determination of materials’ electronic structure^36,37^. While exploiting the exchange–correlation potential in the application of generalized gradient approximation (GGA) structural properties of the materials were determined^35^. Following the Tran and Blaha modified Becke–Johnson potential ^38^, which doesn’t underestimate the bandgaps of the materials unlike the generalized gradient approximation (GGA), the optoelectronic properties of the present alkali (Na)-based niobate (NaNbO_3_) and tantalate (NaTaO_3_) perovskites were determined. Functions based on FP-LAPW having a minimum radius of the muffin-tin sphere (RMT) x K_max_ (which was 8) were used to get the required convergence. The energy loss function was calculated by employing the Fermi’s golden rule and dipole matrix elements^39–41^. For alkali (Na)-based niobate, the RMT values were taken as 2.50, 1.87 and 1.69 a. u. for Na, Nb and O, respectively, while 2.50, 1.92 and 1.65 a. u. were the values respectively for Na, Ta, and O in case of alkali (Na)-based tantalate. An extension in the spherical harmonics within the muffin-tin spheres was made up to the I_max_ = 10, while Fourier expanded charge density was taken to be G_max_ = 12 (a. u.)^−1^. The energy volume curve was fit with Birch–Murnaghan equation providing the structural parameters^42^.

## Results and discussion

### Structural properties

**Figure 1:**
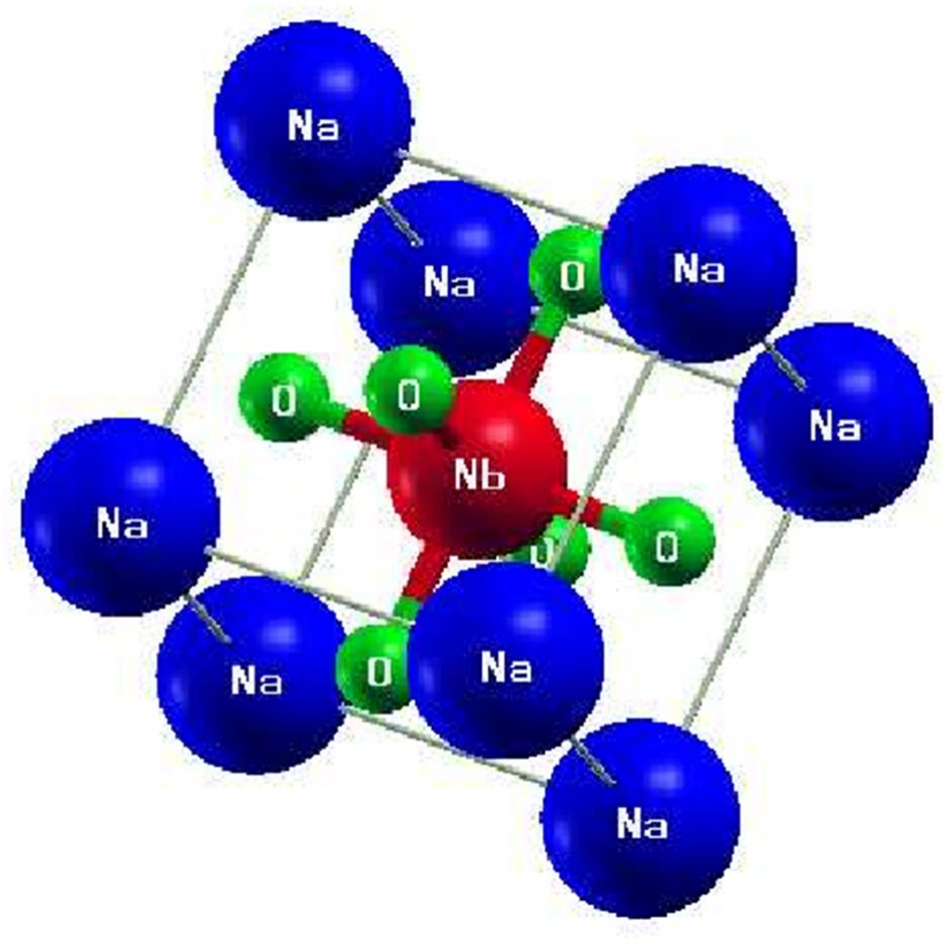
(Color online) A simple cubic structure of alkali (Na)-based niobate (NaNbO_3_): the blue-, red- and green-colored spheres represent sodium, niobium, and oxygen, respectively. The structure of alkali (Na)-based tantalate (NaTaO_3_) is like this one where Na is replaced by Ta.

Figure 1 illustrates a simple cubic structure of one of the two alkali-based perovskite oxides, i.e., NaNbO_3_ with Na residing on-site A while Nb on B where A and B are cations in the general formula of perovskite oxides (ABO_3_). The structure of the other compound, i.e., alkali-based tantalate perovskite oxide (NaTaO_3_) can be estimated like this one where Na is replaced with Ta. As can be seen from Fig. 1, the atom residing on site A, i.e., Na, is larger than that placed on B (Nb), which is compatible with the general structure of the perovskite oxides. With a space group of pm-3 m (221), in the simple cubic structure of the compound, the sodium cation coordinates with 12 oxygen anions while niobium with 6. The coordinates of Na and Nb are respectively (0.0, 0.0, 0.0) and (0.5, 0.5, 0.5) while those of oxygen are (0.0, 0.5, 0.5), (0.5, 0.0, 0.5) and (0.5, 0.5, 0.0).

To carry out all characterizations of material, volume optimization of its unit cell is imperative as a different unit cell results in changes in the properties of the material. To get the volume optimization, we use the Birch–Murnaghan equation of state for fitting. Illustrating energy depending on volume, the Birch Murnaghan fit is employed to interpolate our determined points analytically for finding the ground-state parameters like lattice constant a_0_, and the bulk modulus B.

**Figure 2:**
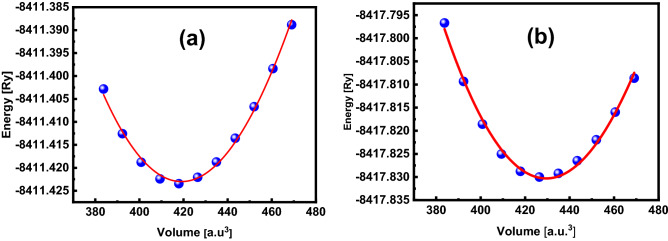
Volume-optimization curve of alkali (Na)-based (**a**) niobate (NaNbO_3_) and (**b**) tantalate (NaTaO_3_), fitted with Birch–Murnaghan equation.

Figure 2 depicts the volume-optimization curve for simple cubic of alkali (Na)-based niobate (NaNbO_3_) and tantalate (NaTaO_3_) perovskite oxides, which have been achieved by reducing the total energy of a unit cell by changing the volume. The total energy for which the volume is minimum is known as ground state energy (E_0_), while the volume is ground state volume (V_0_). A material with optimized energy is expected to have a stable structure.

Table 1 lists all structural parameters such as optimized lattice constants (a_0_), ground state energy(E_0_), volume (V_0_), and the bulk modulus (B_0_), obtained while optimizing the volume of the materials. Since NaTaO_3_ has more minimum energy than NaNbO_3_ and the energy-volume curve for NaTaO_3_ is sharper than that for NaNbO_3_, the former is predicted to have more structural stability than the latter (NaNbO_3_). These optimized parameters, particularly the lattice constants (a_0_), were used to carry out further calculations.

**Table 1:** The structural parameters, i.e., lattice constant (a_0_), optimized volume (V_0_), Bulk modulus (B_0_), pressure derivative (B′) and ground-state energy (E_0_), of ternary alkali (Na)-based niobate (NaNbO_3_) and tantalate (NaTaO_3_) perovskites, obtained by the Birch–Murnaghan fitting to the energy-volume curves.

Structural parameters	NaNbO_3_	NaTaO_3_
a_0_ (Å)	3.95	3.98
V_0_ (a.u^3^)	419.11	429.48
B_0_ (GPa)	183.59589	191.64
B′	− 647.32	− 75.64
E_0_ (Ry)	− 8411.42	− 8417.83

**Figure 3:**
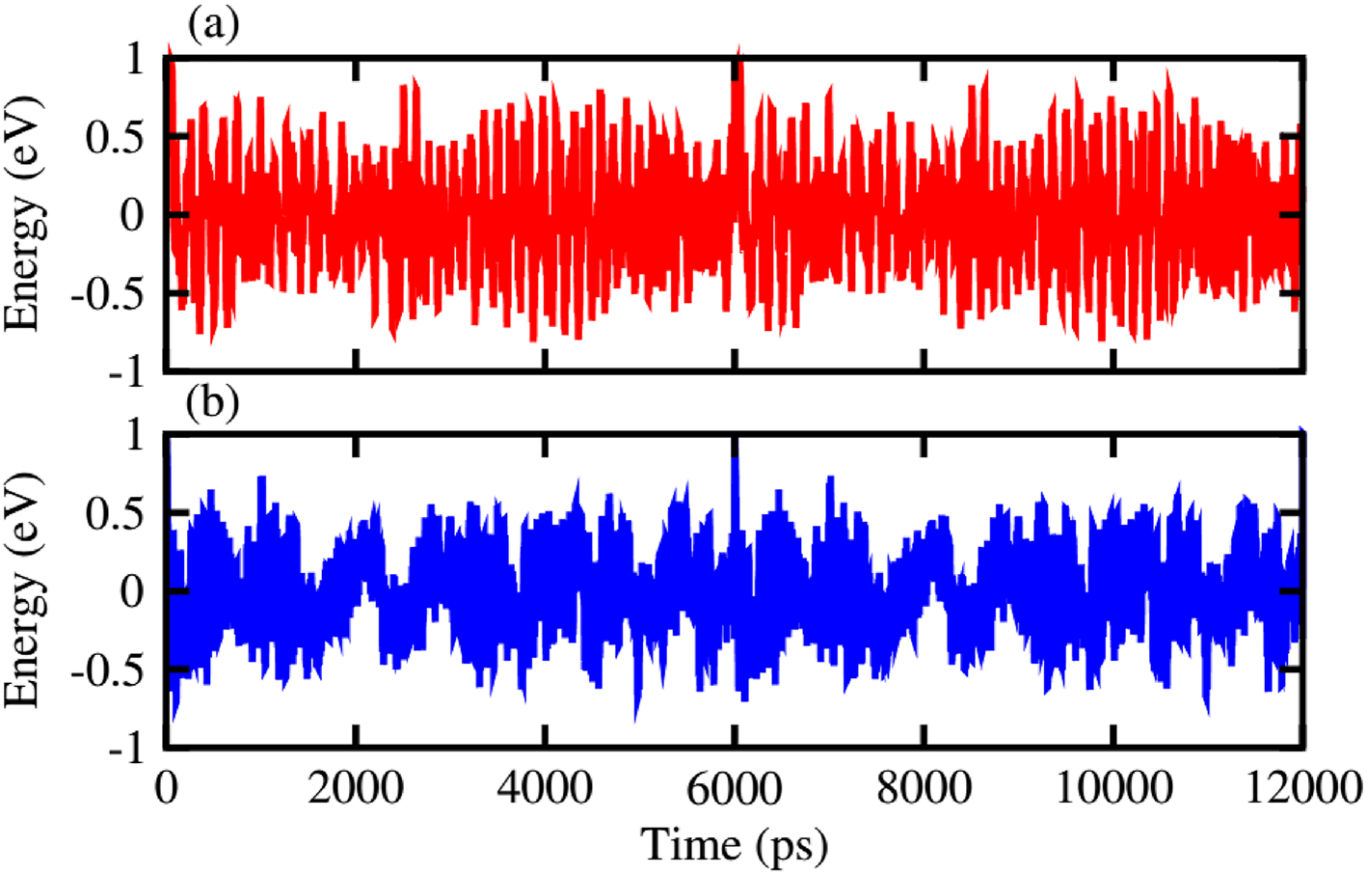
Thermal stability of alkali (Na)-based (**a**) niobate [NaNbO_3_] and (**b**) tantalate [NaTaO_3_] perovskites.

To determine the thermal stability of NaNbO_3_ and NaTaO_3_, we have carried out their ab-initio molecular dynamics simulations (AIMD)^43^ at room temperature for 6 ps with a time interval of 1 fs, as shown in Fig. 3. Figure 3 exhibits that there is very small fluctuation in energy and both perovskites retain their geometries without any structural distortion, hence confirming that NaNbO_3_ and NaTaO_3_ are stable at room temperature.

### Electronic properties

**Figure 4:**
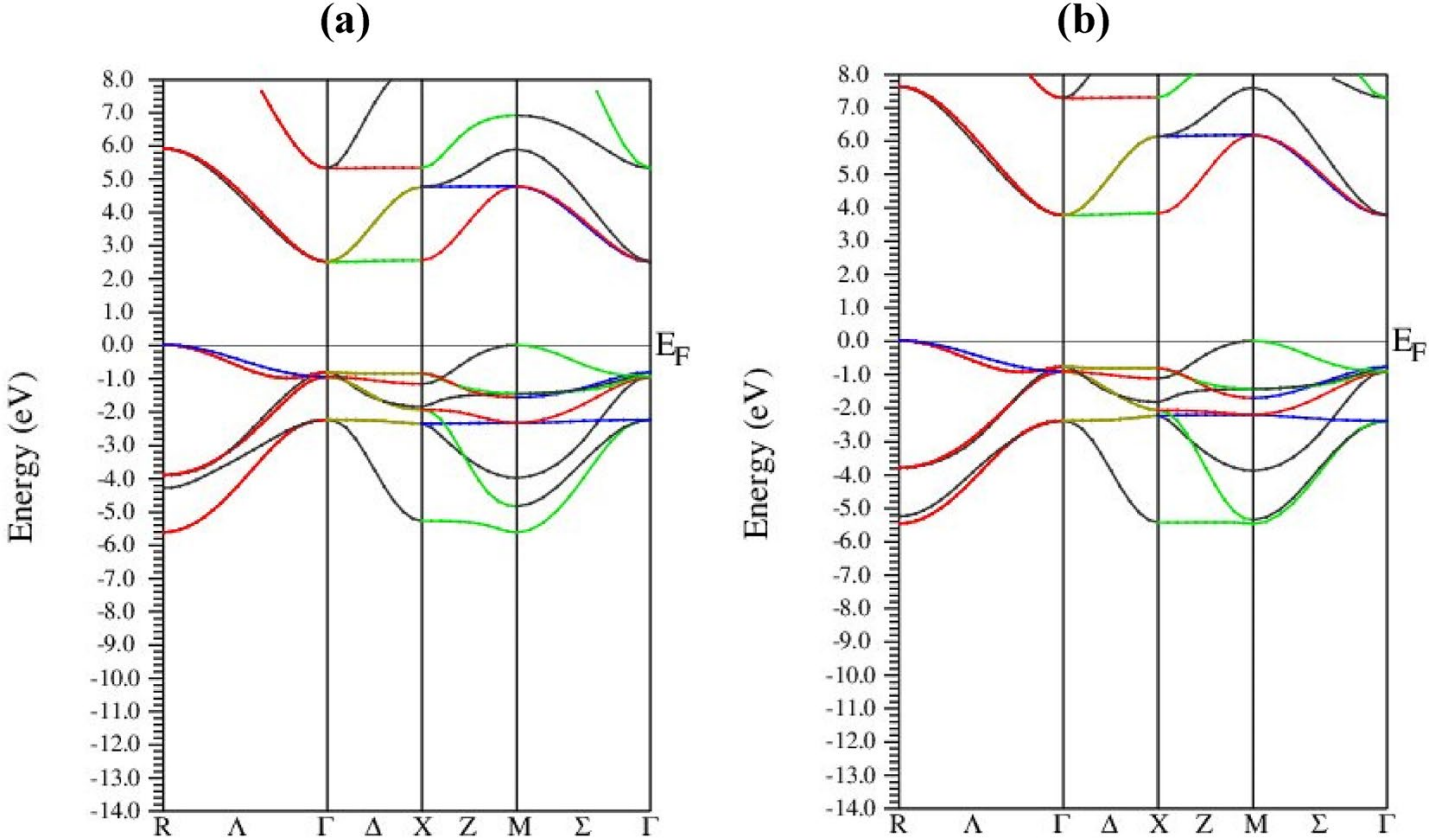
Band-structure diagrams for alkali (Na)-based (**a**) niobate (NaNbO_3_) and (**b**) tantalate (NaTaO_3_).

Band structure describes the behavior of electrons and their state in a given material^44^ by providing information regarding their energy and momentum. Both electrical and optical properties, i.e., how a material responds when subjected to electromagnetic radiations, of material are functions of electronic state and behavior. The optical properties include dielectric functions, electrical conductivity, absorbance, refractive index, and reflectivity. For the determination of electronic characteristics of the studied alkali (Na)-based niobate (NaNbO_3_) and tantalate (NaTaO_3_) perovskite oxides, we exploited modified Becke–Johnson (mBJ) potential to find their band-structure profile. Figure 4 illustrates the band profile of the simple cubic niobate and tantalate oxides perovskites: the first Brillion zone with high symmetry is shown as well. The valence and conduction bands overlap with each other at zero energy, known as the Fermi energy. mBJ was employed for the calculation of results, which demonstrate that these results are in accord with those obtained by FP-LAPW^41^.

Both parts of Fig. 4, i.e., (a) and (b), show that the valence band minima lie at M point in the Brillion zone while at Γ point, the conduction band maxima occur: both niobate and tantalate perovskite oxides exhibit the indirect semiconducting nature. The bandgap of NaNbO_3_ and NaTaO_3_ turned out to be 2.5 eV and 3.8 eV, respectively, that is the latter is a more wide-bandgap semiconductor than the former. The electronic characteristics also are determined by investigating the density of states (DOS) whose results are to be compatible with those of the band structure. The DOS indicates the number of states in which the electrons are allowed to reside at a particular energy. In other words, it tells us how many numbers of electronic levels per unit volume per unit of energy are there.

**Figure 5:**
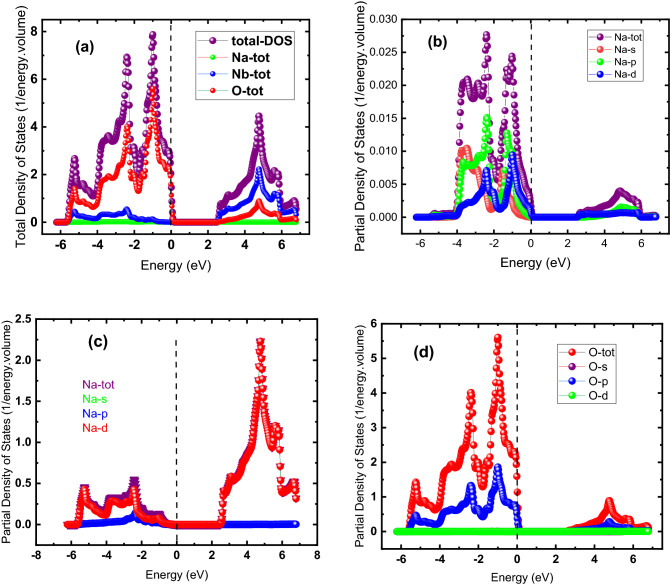
(Color online) DOS for NaNbO_3_ oxide perovskites (**a**) TDOS and PDOS for (**b**) Na, (**c**) Nb and (**d**) O.

**Figure 6:**
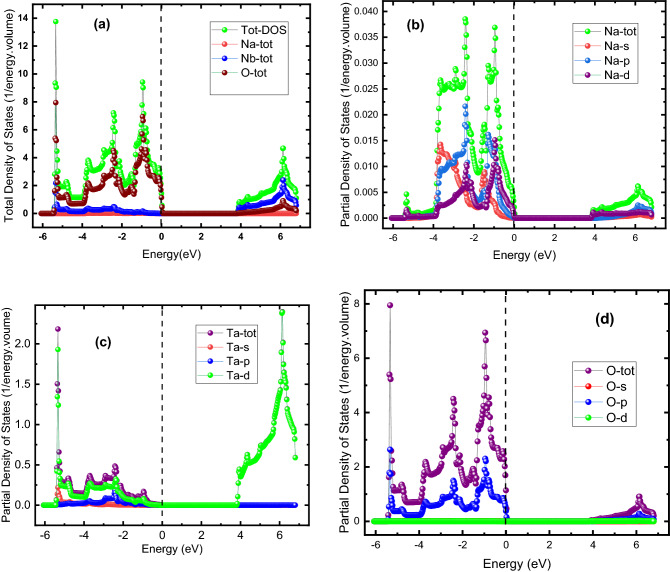
DOS for NaTaO_3_ oxide perovskites (**a**) TDOS and PDOS for (**b**) Na, (**c**) Nb and (**d**) O.

We determined the total and partial density of states, i.e., (TDOS) and (PDOS), respectively, for both materials. The TDOS shows the total DOS of material and the contribution each of its constituents makes to the TDOS while the contribution of subshells, such as p, s, and d, to the density of states of an individual atom, is shown by PDOS. Both TDOS and PDOS for NaNbO_3_ and NaTaO_3_ are respectively shown in Figs. 5 and 6. As estimated from the band-structure profile in Fig. 4, DOS compatibly reveals both materials to be semiconductors in nature, although their nature, i.e., direct, or indirect, cannot be estimated by DOS as can be done by the band-profile structure in Fig. 4. Figure 5 shows the DOS for NaNbO_3_ perovskite oxides: parts (a) and (b), (c) and (d) illustrate TDOS and PDOS for Na, Nb, and O, respectively. Figure 5a exhibits that TDOS in the valence band of the NaNbO_3_ is predominantly contributed by the oxygen atoms followed by a contribution by Na and Nb, minorly. The absence of DOS across the Fermi energy state shows the semiconducting nature of the compound with a bandgap energy of 2.5 eV, consistent with the value revealed by its band structure in Fig. 4a. In the valence band of Fig. 5a, the contribution of Nb to the DOS between 2.6 and 6.5 eV exceeds those of the other two atoms, i.e., Na and O. The relative contribution of these atoms to the TDOS lies in their electronic configuration: the Na, Nb, and O have electronic configurations as [Ne]3s^1^, [Kr] 4d^4^5s^1^ and [He] 2s^2^ 2p^4^, respectively. Due to a greater number of unpaired valence electrons (three) than in the Na and Nb each with one valence electron, oxygen causes a greater contribution than Na and Nb. Similarly, the relative contribution of s, p, and d subshells to the partial density of states of Na, Nb, and O can be explained based on the number of unpaired valence electrons: Due to one electron in s subshell in the [Ne]3s^1^ electronic configuration of Na, the s-subshell contributes more to the PDOS of individual oxygen than the other subshells. The TDOS of Na in Fig. 5c is.

predominantly caused by its d-subshell PDOS followed by a contribution from the p-subshell while the contribution of its s-subshell is negligible. The TDOS for NaTaO_3_ is exhibited in Fig. 6a, while (b), (c), and (d) parts of Fig. 6 illustrate the PDOS of individual Na, Ta, and O atoms, respectively. Figure 6a shows that the majority of the TDOS occurs in valence bands ranging from -5.8 to 0 eV and which are mainly caused by the PDOS of the oxygen atom. The zero density of states from 0 to 3.8 eV in the conduction band indicates the bandgap of this material to be 3.8 eV, consistent with the result of the band structure in Fig. 4b. Figure 6b shows the PDOS of Na in NaTaO_3_, where the TDOS of Na is contributed more by s- and p-subshells of Na in the valence band, while in the conduction band there is less population of states. The PDOS of Ta is caused by its d-subshell while there is a smaller contribution of its other subshells, i.e., s and p, and the states in the valence band are denser than in the conduction band (Fig. 6c). Figure 6d exhibits that lies mostly in the valence band, the TDOS is mainly caused by the p-subshell of O while the contribution from other subshells is negligible.

### Elastic properties

To explore the elastic properties of a material, the determination of elastic constants is important as they establish a material’s response to external forces and provide insight into the mechanical characteristics of the material. Thus, such constants reveal how material is mechanically stable and tough. At zero pressure, the desired constants were computed where the components of the stress tensor for small strains were calculated, and the energy was applied according to the lattice strain which kept the volume constant^44^. Interfaced with WIEN2K and developed particularly for cubic systems, the IRelast package was used to determine the elastic constants. In the case of cubic structure, there is only three total independent elastic constants C_ij_, i.e., C_11_, C_12*,*_ and C_14_. Equations (1)–(7)^45,46^ were used to find out various elastic properties of the materials.

1 $$B = ({C}_{11} + 2{C}_{12})/3$$

2 $${G}_{v} = ({C}_{11} - {C}_{12} + 3{C}_{44})/5$$

3 $${G}_{R} = 5{C}_{44}({C}_{11} - {C}_{12})/(4{C}_{44} + 3{C}_{11} - {C}_{12})$$

4 $$G = ({G}_{v} + {G}_{R})/2$$

5 $$E = 9BG/(G + 3B)$$

6 $$V = (3B-2G)/2(G + 2B)$$

7 $$A = 2{C}_{44}/({C}_{11}-{C}_{12})$$

whereas, ${G}_{R}$ and ${G}_{v}$ are Reuss and Voigt shear moduli respectively^47^, ${C}_{11}$, ${C}_{12}$ and ${C}_{44}$ are elastic stiffness coefficients^46^, $V$ is the Poisson’s ratio, B is bulk modulus, $A$ is Zener’s constant that predicts the anisotropy, $\Delta V$ is change in volume while $p$ is pressure.

Table 2 lists the measured elastic parameters for both materials of sodium niobate (NaNbO_3_) and sodium tantalate (NaTaO_3_).

**Table 2:** Elastic parameters for the sodium niobate (NaNbO_3_) and sodium tantalate (NaTaO_3_): elastic stiffness coefficients (${C}_{11}$, ${C}_{12}$ and ${C}_{44}$), bulk modulus (B) and Zener’s constant (A).

Elastic parameters	NaNbO_3_	NaTaO_3_
${C}_{11}$(GPa)	407.12	272.62
${C}_{12}$(GPa)	69.08	51.28
${C}_{44}$(GPa)	72.80	56.42
*B* (GPa)	181.76	125.06
$A$	0.43	0.50
${G}_{v}$(GPa)	2.49	2.12
${G}_{R}$(GPa)	338.34	221.34
$G$(GPa)	233.5	159.1
$E$(GPa)	490.52	335.11
$V$	0.1	0.1
*B*/*G*	0.77	0.78

${G}_{v}$, ${G}_{R}$ and $G$ are Voigt shear modulus, Reuss shear modulus, and mean of ${G}_{v}$ and ${G}_{R}$, respectively. E is the Elastic modulus, V is the Poisson’s ratio, B/G is the ratio of bulk to shear moduli. The elastic stiffness coefficients and other constants as well as pressure are in GPa and volume in bohr3.

For mechanical stability of cubic crystals, the elastic constants are to be related with each other such as C_11_–C_12_ > 0 and C_11_ > 0, C_44_ > 0, C_11_ + 2C_12_ > 0 and B > 0^45^. Since the studied materials obey these conditions, they are suggested to be mechanically stable. From Table 2, it is evident that NaNbO_3_ has larger elastic stiffness coefficients suggesting strong ability of the material to withstand compressive as well as shear stresses. On the other hand, NaTaO_3_ has the lower bulk modulus thus suggesting weaker ability to resist change in volume. It can also be seen that NaNbO_3_ has a larger modulus of rigidity G than that of NaTaO_3_ suggesting stronger ability to resist change in the shape. The values of A show that none of the compound is perfectly isotropic and both are half isotropic. Interestingly, the materials’ B/G ratio of the materials is larger than 1.75 for NaNbO_3_ while smaller for NaTaO_3_. The B/G larger than 1.75 suggests ductile nature of the NaNbO_3_ while the smaller suggests brittleness of the NaTaO_3_^48^.

## Optical properties

### Dielectric function

**Figure 7:**
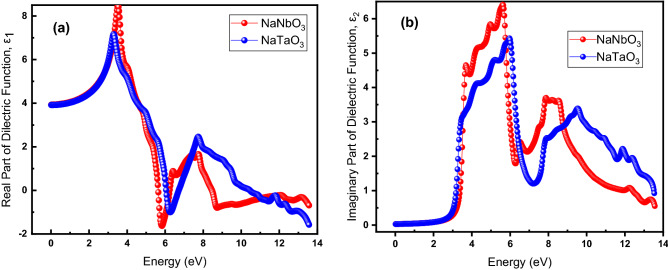
(Color Online) (**a**) real and (**b**) imaginary parts of dielectric function for NaNbO_3_ (red) and NaTaO_3_ oxide perovskites.

For numerous optical properties of NaNbO_3_ and NaTaO_3_, the determination of dielectric function is instructive. How the electromagnetic waves propagate when the medium for them is changed and the interaction between phonons and electrons are described by the complex dielectric function. Given by Eq. (8),

8 $$\varepsilon \left( \omega \right) = \varepsilon_{1} \left( \omega \right) + i\varepsilon_{2} \left( \omega \right),$$

where the ${\varepsilon }_{1}\left(\omega \right)$ and ${\varepsilon }_{2}(\omega )$ are respectively real and imaginary parts, the dielectric function’s determination is imperative for the optical properties of NaNbO_3_ and NaTaO_3_. Figure 7 exhibits the dielectric function for both NaNbO_3_ and NaTaO_3_ where part (a) of Fig. 7 illustrates the real part [*ε*_1_(ω)], while the imaginary part, *ε*_2_(ω), is shown in its part (b): the real part elucidates how the photons are dispersed in the material and the degree of polarization. The *ε*_1_(ω) initially increases with increasing energy while reaching its maximum values at 3.40 and 3.27 eV for NaNbO_3_ and NaTaO_3_, respectively, followed by a dramatic drop to the negative values at 5.88- and 6.34-eV energy, respectively, while fluctuating afterward for both materials. The static dielectric function, *ε*_1_(0), for both materials is found to be the same, i.e., 3.87. Figure 7b illustrates that the *ε*_2_(ω) starts increasing from zero at around 3.0 eV, which is an absorption edge, and reaches its maximum value of 6.41 at 5.69 eV and 5.41 at 5.98 eV for NaNbO_3_ and NaTaO_3_, respectively, indicating that the absorption is maximum in the ultra-violet (UV)-visible (Vis) region. This drops abruptly to 6.17 and 7.26 eV respectively and afterward starts fluctuating. The absorption in the UV–Vis region makes these materials promising for applications in the optoelectronic devices such as light emitting diodes (LEDs).

### Optical conductivity

**Figure 8:**
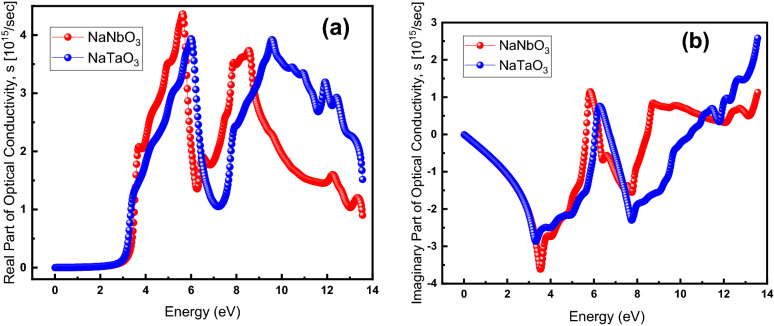
(Color Online) (**a**) real and (**b**) imaginary parts of optical conductivity for NaNbO_3_ (red) and NaTaO_3_ oxide perovskites.

Figure 8 compares the optical conductivity of NaNbO_3_ and NaTaO_3_: the real and imaginary parts of the conductivity are illustrated in parts (a) and (b), respectively.

The real part of optical conductivity (Fig. 8a) shows that the conductivity is maximum at 5.65 and 6.02 eV for NaNbO_3_ and NaTaO_3_, respectively. The conductivity of NaNbO_3_ at lower energy than that of NaTaO_3_ is attributed to the lower bandgap energy of the former than the latter as revealed by Fig. 4, i.e., due to the lower bandgap energy, the electrons are moved from the valence band to the conduction band more easily (at lower energy), thus contributing to the conductivity. Figure 8b shows the imaginary part of the optical conductivity, describing the screening of the applied field. The imaginary part of the optical conductivity decreases from 0 to − 2.91 in the 0–3.25-energy range for NaTaO_3_ while for the NaNbO_3_, it decreases from -3.63 in the energy ranging from 0 to 3.55. They respectively reach their maximum value at 6.26 and 5.77 followed by a dramatic drop in both. After around 7.7 eV, however, its starts fluctuations with no clear trend for both materials.

### Refractive index and reflectivity

**Figure 9:**
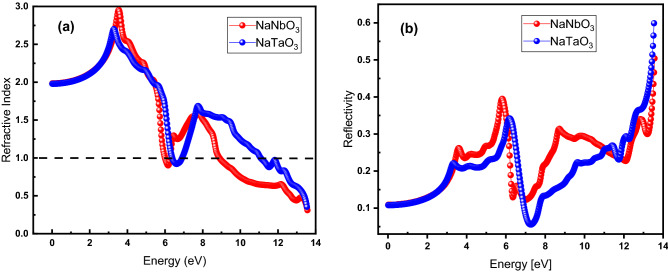
(Color Online) (**a**) refractive index and (**b**) reflectivity as a function of energy for NaNbO3 (red) and NaTaO3 (blue) oxide perovskites.

Figure 9A shows the determined refractive index for NaNbO_3_ (red) and NaTaO_3_ (blue) in the energy range of 0–14 eV, where the static refractive index is 1.97 for both materials. The curves for both materials while coinciding with each other start increasing with increasing energy and get to the maximum values of 2.67 at 3.30 eV and 2.9 at 3.53 eV for NaTaO_3_ and NaNbO_3_, respectively.

The higher refractive index for NaNbO_3_ than that for NaTaO_3_ suggests that the photons are more retarded, resulting from the electronic polarization while traveling through the medium of the former. The degree of electronic polarization depends upon the size of the constituent atoms of material: the Ta atom with a larger size produces a higher polarization, leading to the higher retardation (slower velocity) of the photon inside the material, and, hence, lower refractive index. It reduces to 0.82 at 6.14 and 0.92 at 6.67 eV for NaNbO_3_ and NaTaO_3_, respectively. This is followed up with increasing energy before dropping down at higher energies (7.87 – 14 eV). Figure 9b exhibits reflectivity of the NaNbO_3_ (red) and NaTaO_3_ (blue) in the energy range of 0-to-14 eV. For both materials, the zero-frequency reflectivity is found to be matching with the value of 0.10, which starts increasing with increasing energy of photons and get the maximum value of 0.4 at 5.73 eV and 0.34 at 6.15 eV for NaNbO_3_ and NaTaO_3_, respectively. The lower value for reflectivity at low energy (in the range of bandgap of the material) is attributed to the transparency of the materials to the incidence of photons. This makes these materials promising for applications in lens fabrication.

### Extinction coefficient

Demonstrating the degree of light absorption by a material, the extinction coefficient, $k\left(\omega \right)$, was calculated by Eq. (9):

9 $$k\left( \omega \right) = ~\left( {\frac{1}{2}\left[ {\sqrt {\varepsilon _{1} ^{2} \left( \omega \right) + \varepsilon _{2} ^{2} \left( \omega \right)} - \varepsilon _{1} \left( \omega \right)} \right]} \right)^{{1/2}}$$

**Figure 10:**
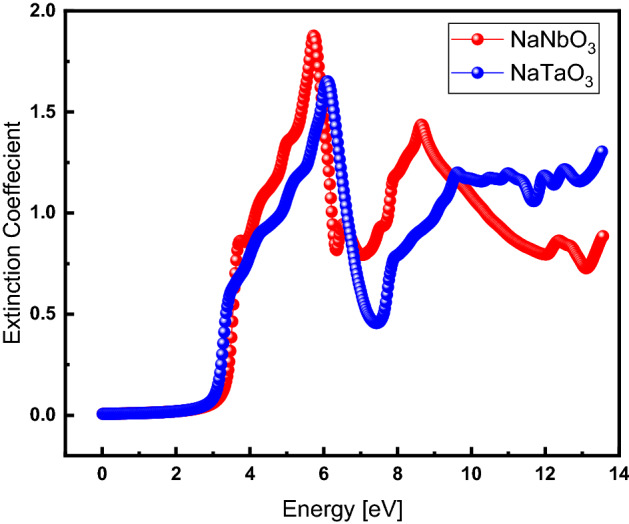
(Color Online) Extinction coefficient as a function of energy for NaNbO_3_ (red) and NaTaO_3_ (blue) oxide perovskites.

Figure 10 exhibits the extinction coefficient for both NaNbO_3_ and NaTaO_3_, represented by red- and blue-colored data, respectively, where it is zero for the photon energy ranging from 0 to 3.0 eV and starts increasing with further increasing energy. The extinction coefficient gets to its maximum value at 5.87 and 6.17 eV for NaNbO_3_ and NaTaO_3_, respectively. This decreases at higher energy followed by fluctuations with increasing energy beyond 7.5 eV.

### Energy-loss function

**Figure 11:**
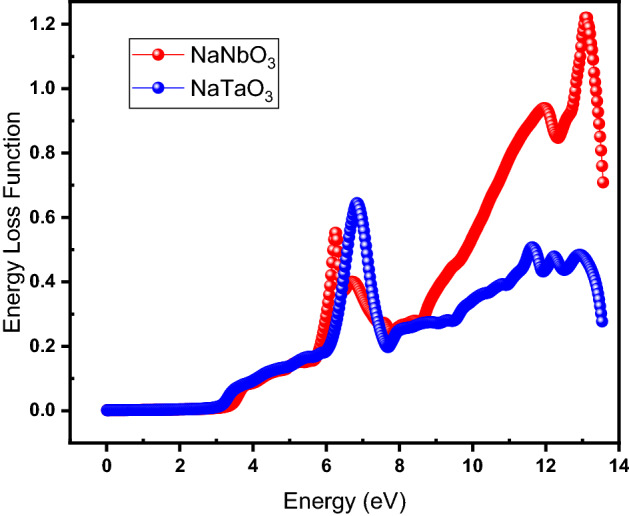
(Color Online) Energy loss function as a function of energy for NaNbO_3_ (red) and NaTaO_3_ (blue) oxide perovskites.

Describing the intra-band, inter-band, and plasmon interdependencies, the energy-loss function for both NaNbO_3_ and NaTaO_3_ is shown in Fig. 11, represented by red and blue data points, respectively. The energy-loss function also describes the energy lost by an electron while moving fast through a material. Figure 11 shows that the energy-loss function for both materials is zero for the energy of the photon ranging from 0 to 3.18 eV and starts increasing with increasing photon energy while peaking at 6.24 and 6.86 eV for NaNbO_3_ and NaTaO_3,_ respectively. At higher energies, i.e., beyond 7.75 eV, energy loss hikes up for NaNbO_3_ while there is no significant variation in it for NaTaO_3_.

## Conclusion

In conclusion, we studied the structural and optoelectronic properties of alkali (Na)-based niobate (NaNbO_3_) and tantalate (NaTaO_3_) perovskites while using the DFT, embedded in WIEN2K. Fitted with the Birch–Murnaghan equation of state, the energy-volume curve demonstrated the structural stability of both materials. The ab-initio molecular dynamics simulations (AIMD) showed that compound both perovskites retain their geometries without any structural distortion, hence confirming that they are stable at room temperature. By exploiting the mBJ method, the calculated electronic properties revealed the semiconductor nature of the materials with indirect bandgap energy of 2.5 and 3.8 eV for NaNbO_3_ and NaTaO_3_, respectively. The computed total and partial density of states also exhibited the same values of bandgap energy. The optical properties such as dielectric function, optical conductivity, reflectivity, refraction, energy loss function, and extinction coefficient exhibited that these materials have potential application in UV devices. It was also found that these materials are mechanically stable. NaNbO_3_ exhibited stronger ability to bear the compressive and shear stresses and resist change in shape while NaTaO_3_ showed poor ability to oppose the change in volume. None of the compound was found to be perfectly isotropic and NaNbO_3_ and NaTaO_3_ showed ductility and brittleness, respectively. Both materials exhibited transparency to the incident photon at low energy and absorption and optical conduction in the UV–Vis region, making them promise for optoelectronic applications. We hope that for further investigations, this study will draw the attention of experimental studies.

## Data Availability

Data can be provided on request made to the first author, Shaukat Ali Khattak.
